# The Complete Genome Sequence of *Cyanopsitta spixii,* the Spix’s Macaw

**DOI:** 10.56179/001c.37839

**Published:** 2022-08-20

**Authors:** Taylor Hains, Stacy Pirro, John Bates, Shannon Hackett

**Affiliations:** 1 Negaunee Integrative Research Center, Field Museum of Natural History; 2 Iridian Genomes

**Keywords:** genome, parrot, endangered, conservation

## Abstract

The Spix’s Macaw (*Cyanopsitta spixii*) is a critically endangered parrot that was once endemic to Brazil. We present the whole genome sequence of this species. Illumina sequencing was performed on a genetic sample from a single captive individual. The reads were assembled using a de novo method followed by a series of references from related species for finishing. The raw and assembled data is publicly available via Genbank: Sequence Read Archive (SRR15037507) and Assembly (GCA_024336845).

## Introduction

The Spix’s Macaw is a medium-sized parrot with grayish-blue plumage. Males are slightly larger than females but otherwise identical in appearance.

The species was endemic to riparian Caraibeira (*Tabebuia aurea*) gallery woodlands in the drainage basin of the Rio São Francisco within the Caatinga of northeastern Brazil. It had a very restricted natural habitat along several small rivers. It fed primarily on seeds of two species of Euphorbiaceae, the dominant vegetation of the Caatinga (Barros et al. 2012). Due to deforestation in its limited range and specialized habitat, the bird was rare in the wild throughout the twentieth century. The IUCN conducted several years of survey attempting to locate living wild populations, but it was officially declared extinct in the wild in 2019.

## Methods

The genetic sample was provided from San Diego Zoo Wildlife Alliance’s Wildlife Biodiversity Bank by the Conservation Genetics Team.

DNA extraction was performed using the Qiagen DNAeasy genomic extraction kit using the standard process. A paired-end sequencing library was constructed using the Illumina TruSeq kit according to the manufacturer’s instructions. The library was sequenced on an Illumina Hi-Seq platform in paired-end, 2 × 150bp format. The resulting fastq files were trimmed of adapter/primer sequence and low-quality regions with Trimmomatic v0.33 (Bolger, Lohse, and Usadel 2014). The trimmed sequence was assembled by SPAdes v2.5 (Bankevich et al. 2012) followed by a finishing step using Zanfona (Kieras, O’Neill, and Pirro 2021).

## Results

The genome assembly yielded a total sequence length of 1,092,480,392 bp over 53,615 scaffolds.

## Data Availability

Raw and assembled data is publicly available via GenBank: raw genome data https://trace.ncbi.nlm.nih.gov/Traces/sra/?run=SRR15037507 assembled genome https://www.ncbi.nlm.nih.gov/assembly/GCA_024336845
